# Eliciting renal tenderness by sonopalpation in diagnosing acute pyelonephritis

**DOI:** 10.1186/s13089-016-0056-6

**Published:** 2017-01-03

**Authors:** Jeremy S. Faust, James W. Tsung

**Affiliations:** 1 Department of Emergency Medicine, Harvard Medical School, Brigham and Women’s Hospital, 10 Vining Street, Neville House, Boston, MA 02115 USA; 2 Department of Emergency Medicine and Division of Emergency Ultrasound, Icahn School of Medicine, Mount Sinai Hospital, New York, NY USA

**Keywords:** Emergency medicine, Pyelonephritis, Point-of-care ultrasonography, Sonopalpation

## Abstract

Diagnosing acute pyelonephritis relies on the combination of historical, physical, and laboratory findings. Costovertebral angle tenderness is important, although its accuracy is unknown. Point-of-care ultrasound-guided palpation (sonopalpation) may aid clinicians in localizing pain to discrete anatomic structures in cases of suspected acute pyelonephritis lacking classic features. We describe three low-to-moderate pre-test probability cases wherein maximal tenderness was elicited by renal sonopalpation, aiding in the diagnosis of acute pyelonephritis. In a fourth case, absence of renal tenderness to sonopalpation in a patient exhibiting typical acute pyelonephritis features led to an alternate diagnosis. Therefore, renal sonopalpation may be useful in confirming or refuting suspected cases.

## Background

No single finding is diagnostic of acute pyelonephritis. The decision to treat relies on a combination of history, physical examination, and laboratory findings. Establishing the diagnosis in part by eliciting costovertebral angle tenderness is a standard physical examination maneuver, first described in 1884 by American surgeon Murphy [1]. However, little data exist regarding its diagnostic accuracy [2]. Because body habitus of patients can vary greatly, it can be challenging to accurately determine the location of the costovertebral angle. The combination of ultrasound imaging and physical examination to localize pain and correlate it to a specific visualized anatomic structure has been termed “sonopalpation” and may achieve higher diagnostic accuracy than physical examination alone [3].

Point-of-care ultrasound has been shown to aid in source identification in patients with suspected sepsis [4]. However, the role of point-of-care ultrasound in identifying low-to-moderate pre-test probability cases of pyelonephritis has not been investigated. Doing so may lead to more accurate and rapid source identification in potentially septic patients, thereby promoting antibiotic stewardship.

In this study, we describe a series of three patients with acute pyelonephritis where costovertebral angle tenderness was equivocal, but sonopalpation definitively localized maximal pain and tenderness to the kidneys. In a fourth patient in which acute pyelonephritis was initially suspected, nontender kidney sonopalpation eventually led to an alternative diagnosis. In each of these cases, a second sonologist observer independently confirmed the presence or absence of renal tenderness to sonopalpation, with perfect inter-operator agreement.

## Main text

### Case 1

A 21-year-old woman with a past medical history of one uncomplicated urinary tract infection with a pan-sensitive urine culture presented to the emergency department (ED) with 2 days of new right-sided abdominal pain, noting some radiation to back. The patient had two episodes of nonbloody/nonbilious emesis the day before the encounter, but was able to tolerate liquids on day of the visit. The patient denied dysuria or other urinary changes. Vital signs were notable for a temperature of 100.9 °F and a heart rate of 130 bpm. Examination elicited right upper quadrant tenderness and right lateral abdominal tenderness, but no rigidity or rebound tenderness. The patient was nontender elsewhere, including the suprapubic region. While performing a right upper quadrant point-of-care ultrasound to assess for biliary pathology, a negative sonographic Murphy sign was noted, while maximal tenderness was found directly over the ipsilateral kidney, in all orientations [5]. Tenderness increased with increasing pressure applied by the ultrasound probe. There were no other notable kidney abnormalities and the immediately adjacent areas were nontender to sonopalpation. Subsequent urinalysis testing was remarkable for nitrites and leukocyte esterase. The patient was successfully treated as an outpatient with cephalexin. At one month follow-up, the patient reported resolution of symptoms after the antibiotic course.

### Case 2

A 19-year-old female with a history of recurrent but uncomplicated urinary tract infections presented to the ED with dysuria for 3–4 days and bilateral flank, back, and lateral abdominal pain, worse on the left. The patient noted nausea and one episode of vomiting at home. In the ED, the patient had normal vital signs but was uncomfortable and unable to tolerate oral pain medications. On physical examination, the patient was tender to palpation in the left mid-lateral abdomen, left lower back, and left costovertebral region. Point-of-care ultrasound delineated these non-specific findings and demonstrated maximal tenderness with direct sonopalpation over the left kidney (which demonstrated no hydronephrosis.) The patient was subsequently admitted for intravenous ceftriaxone, fluids, antiemetics, and pain control. Urinalysis revealed large leukocytes and a urine culture grew ampicillin-resistant but otherwise pan-sensitive *Escherichia coli*. Blood testing revealed no electrolyte abnormalities or leukocytosis, though an 80% neutrophil predominance was noted. A non-contrast computed tomography of the abdomen revealed no renal stones or perinephric stranding. A radiology suite ultrasound showed no hydronephrosis. The patient improved with antibiotic treatment and was discharged without complication on the 2nd day of hospitalization.

### Case 3

A 37-year-old woman with no past medical history presented to the ED with a chief complaint of headache, body pain, and minimal dark vaginal discharge. In triage, she was febrile to 102.8 °F and tachycardic to 138 beats per minute and thus triggered a sepsis alert. She denied dysuria or pelvic pain. She also denied symptoms consistent with upper respiratory infections or gastrointestinal infections. She exhibited no altered mental status, neck pain, stiffness, or rash. On review of symptoms, she complained of mild right mid back pain. Her examination was unremarkable, including a normal pelvic examination. While she denied oliguria, the patient was initially unable to provide urine. A chest X-ray was normal. Initial lactate was normal at 1.8 mmol/L. Given no clear source of infection, imipenem-cilastatin was ordered. However, before the antibiotic was administered, a point-of-care ultrasound was performed demonstrating unequivocal and maximal tenderness to the right kidney on sonopalpation. At this point, the patient was able to produce a small amount of urine, and point-of-care urinalysis demonstrated 3+ blood and small leukocyte esterase. Given this, the diagnosis of pyelonephritis was made and ceftriaxone was administered instead of imipenem–cilastatin. Blood tests resulted as unremarkable, and urine culture and gonorrhea and chlamydia tests results were pending. The patient remained tachycardic in the 110 s for two more hours and her oral temperature improved to 99.8 °F. The patient was admitted to the ED observation unit for further doses of intravenous ceftriaxone where she improved dramatically over the subsequent 12 h and was discharged. She completed a course of oral antibiotics with excellent response. On follow-up, the urine culture grew *E. coli* and gonorrhea and chlamydia tests were negative.

### Case 4

A 25-year-old obese woman with an intrauterine contraceptive device in place for two years presented to the ED with 7 days of dysuria and “colicky” nonradiating suprapubic pain which worsened one day earlier. She complained of a new fever and lower back pain in the past day, but no other infectious symptoms other than one year of occasional mild white vaginal discharge and trace vaginal spotting associated with the end of her menstruation. She stated a monogamous sexual relationship with no new partners. In triage, her temperature was 100.8 °F with otherwise unremarkable vital signs. On examination, she had moderate suprapubic tenderness. She also had tenderness in the right lower back. Because of obesity, costovertebral angle tenderness could not be definitively established. Subsequent point-of-care ultrasound demonstrated a maximal area of tenderness well inferior to the patient’s right kidney, which was relatively superior and medial, in this patient. A bimanual pelvic examination was then performed, demonstrating mild cervical erythema and localized tenderness in the right adnexal region. An endocervical swab was obtained for gonococcal and chlamydia testing. Urinalysis revealed moderate leukocyte esterase. The patient was empirically treated for pelvic inflammatory disease with intramuscular ceftriaxone and oral doxycycline. She was also treated with oral cephalexin in the case of non-pyuric cystitis. The urine culture grew no organisms. The endocervical swab was positive for chlamydia. The patient was notified and advised to notify her partner. No complications were reported on a subsequent unrelated ED visit four months later.

## Discussion/conclusion

Sonopalpation is a well-established concept [3]. Its best-known application is the “sonographic Murphy sign,” which has 63% sensitivity and 93.6% specificity for ruling-in acute cholecystitis [6]. Other diverse applications have been described, including pelvic inflammatory disease, appendicitis, sialolithiasis, breast cancer, and musculoskeletal disorders [7–12]. In this study, we describe the discovery of a new application for point-of-care sonopalpation: aiding in the diagnosis of pyelonephritis in cases of low-to-moderate pre-test probability cases that deviate from the classic clinical picture.

In our evaluations, sonopalpation was performed using a curvilinear probe. Renal tenderness was assigned only when a patient reported maximal tenderness to sonopalpation directly over the kidney, in comparison to less tender adjacent areas (negative controls.)

Our first case of acute pyelonephritis was discovered fortuitously while attempting to elicit a sonographic Murphy’s sign in a patient with undifferentiated right upper abdominal pain and side pain. The sonographic Murphy sign was absent. However, the point of maximal tenderness was found to be directly over the right kidney. This in conjunction with subsequent urinalysis findings, presence of fever, and tachycardia confirmed the diagnosis of acute pyelonephritis. In the second case, pyelonephritis was suspected when renal sonopalpation was performed, but the patient’s tenderness to standard examination techniques did not localize to the usual costovertebral angle area alone. Therefore, the finding of maximal point tenderness over the left kidney eliminated any diagnostic uncertainty. The third case represented the first instance in which point-of-care sonopalpation of the kidneys altered clinical impression resulting in improved antibiotic stewardship. Given recently lower thresholds to utilize broad-spectrum antibiotics for unexplained sepsis in accordance to clinical guidelines, this case may represent an important future use for renal sonopalpation. Our fourth case was the first instance in which nontender sonopalpation of the kidney in suspected acute pyelonephritis caused us to change our initial diagnostic impression and led to alternate and important findings. This implies that renal sonopalpation may help clinicians reduce diagnostic error in patients whose physical examination may be less reliable, due to body habitus or atypical symptoms.

Taken together, these cases suggest that sonopalpation of the kidney may be a useful and reproducible finding in confirming or refuting the diagnosis of acute pyelonephritis. Kidney sonopalpation may potentially expedite ED care, especially in instances failing to reach an acceptable diagnostic threshold when relying on the tools currently in common use. For example, in the absence of a typical clinical picture combined with equivocal point-of-care urinalysis results (e.g., positive leukocyte esterase only), the resulting post-test probability of these factors may not surpass the treatment threshold for acute pyelonephritis. Positive renal tenderness to sonopalpation may therefore help confirm the diagnosis in such cases. However, these cases represent a very limited experience, and future studies are needed to establish test characteristics and inter-operator agreement of renal sonopalpation for diagnosing acute pyelonephritis as a part of point-of-care renal ultrasound evaluation.
